# Perspectives on setting limits for RF contact currents: a commentary

**DOI:** 10.1186/s12938-018-0434-3

**Published:** 2018-01-15

**Authors:** Richard A. Tell, Christopher A. Tell

**Affiliations:** Richard Tell Associates, Inc., 350 Falcon Ridge Parkway, Suite 103, Mesquite, NV 89027 USA

## Abstract

**Background:**

Limits for exposure to radiofrequency (RF) contact currents are specified in the two dominant RF safety standards and guidelines developed by the Institute of Electrical and Electronics Engineers (IEEE) and the International Commission on Non-Ionizing Radiation Protection (ICNIRP). These limits are intended to prevent RF burns when contacting RF energized objects caused by high local tissue current densities. We explain what contact currents are and review some history of the relevant limits with an emphasis on so-called “touch” contacts, i.e., contact between a person and a contact current source during touch via a very small contact area.

**Results:**

Contact current limits were originally set on the basis of controlling the specific absorption rate resulting from the current flowing through regions of small conductive cross section within the body, such as the wrist or ankle. More recently, contact currents have been based on thresholds of perceived heating. In the latest standard from the IEEE developed for NATO, contact currents have been based on two research studies in which thresholds for perception of thermal warmth or thermal pain have been measured. Importantly, these studies maximized conductive contact between the subject and the contact current source. This factor was found to dominate the response to heating wherein high resistance contact, such as from dry skin, can result in local heating many times that from a highly conductive contact. Other factors such as electrode size and shape, frequency of the current and the physical force associated with contact are found to introduce uncertainty in threshold values when comparing data across multiple studies.

**Conclusions:**

Relying on studies in which the contact current is minimized for a given threshold does not result in conservative protection limits. Future efforts to develop limits on contact currents should include consideration of (1) the basis for the limits (perception, pain, tissue damage); (2) understanding of the practical conditions of real world exposure for contact currents such as contact resistance, size and shape of the contact electrode and applied force at the point of contact; (3) consistency of how contact currents are applied in research studies across different researchers; (4) effects of frequency.

## Introduction

When radiofrequency (RF) currents flow through biological tissues, tissue heating can occur. Such currents, as described in RF safety standards, are designated as “induced” currents or “contact” currents. Induced currents are those that are caused through the interaction of incident RF fields with the body and are typically measured as currents that flow through the feet to ground. In contrast, contact currents are those currents that flow upon contact and at the point of contact between the body and, most usually, an RF energized object. In either case, if the magnitude of RF current is sufficient, tissue heating will occur. In this commentary, we focus on the issue of contact currents since they present the greatest likelihood of overt RF hazards and the fact that exposure limits for contact currents in present day standards are based on relatively little research. This is in stark contrast with the huge database of scientific reports on biological effects related to whole body exposure to modest RF fields. Further, we examine several factors that can lead to uncertainty in previous experimental studies on contact currents. Finally, we outline several recommendations that, in our opinion, should be followed to improve the basis upon which future contact current limits might be developed.

The subject of contact currents as it relates to RF hazards has been a component of RF safety standards for many years. The IEEE first described the matter of RF contact currents in its 1991 standard [1] where it noted that RF currents flowing between a person exposed to electric fields when contacting a grounded object can, in some circumstances, be sufficient to cause highly localized tissue heating at the point of contact. With sustained contact, the contact current can lead to an RF burn. At lower frequencies, typically below about 100 kHz, the tissue heating effect is replaced by electrical stimulation of the nerves in the body, changing from a heating effect to one of electrical shock. Significant documentation of lower frequency phenomena has been published [2] and the matter of contact currents that are relevant to shock effects are not addressed in this review. Here, we provide a brief history of pertinent standards that set limits on RF contact currents and comment on various technical aspects of research studies upon which these limits have been based. A better analysis of contact currents can lead to improved safety standards for exposure to RF energy. Ironically, while RF burns, either via direct contact with energized sources or via an electrical arc to the body (the most serious type of interaction) are undoubtedly the most hazardous aspect of RF exposures, relatively little research has been put into studying this issue. While relatively little focus has been placed on contact currents in the context of safety programs, the matter of accidental burn injuries from high current densities that are a result of electrosurgical procedures has been studied in considerable depth [3, 4].

## Background

Historically, limits on contact currents have been recommended over the years for the stated purpose to “avoid shock and burn hazards”. The International Commission on Non-Ionizing Radiation Protection (ICNIRP) guidelines [5, 6] contain maximum contact current values (reference levels) for radio frequencies of 20 or 40 mA (100 kHz–110 MHz) depending on whether exposure was categorized as general public or occupational, respectively. ICNIRP defined contact current as the current passed into a biological medium via a contacting electrode or other source of current. It is notable that ICNIRP chose to set the upper frequency for contact current limits at 110 MHz which encompasses the FM radio broadcast band. ICNIRP also set reference levels for “limb current” at 45 mA (for public exposure) or 100 mA (for occupational exposure). Interestingly, while ICNIRP specifies that limb currents are to be averaged over any 6-min period to limit local SAR, there is no such specification on averaging time for contact currents. It should be noted that the ICNIRP guidelines have been embraced in the European Directive 2013/35/EU which specifies the minimum health and safety requirements regarding the exposure of workers to the risks arising from physical agents (electromagnetic fields) [7].

The importance of contact currents has usually been associated with shock and burn hazards. Excepting very small duty cycle currents, electrical shock does not happen above about 100 kHz due to the neurological frequency response of the body. In the RF range, the term shock likely best describes the reaction of a person to arcing phenomena wherein high RF voltages can lead to an electrical arc from an energized source to the body surface (non-contact). Arcing, undoubtedly, leads to the most hazardous of all RF biological effects ever observed; tissue destruction is usually the result of such phenomena. For example, the spontaneous reaction of a person to an unexpected sensation of contact current, whether delivered via direct contact or via an electrical arc, has the potential of “triggering” accidents in the work environment. In the context of limiting contact currents, however, the non-contact arcing phenomenon, sometimes referred to as transient spark discharge, is often disregarded in the belief that other controls, such as limiting open circuit voltages, are more relevant and effective. In this commentary, our emphasis is on essentially continuous contact currents.

In the 1991 IEEE standard [1], the contact current maximum permissible exposure (MPE) was described in terms of a “grasping” contact but did not specify what the relevant contact area was. For controlled environments, both the total induced body RMS current and contact currents were to be measured with an averaging time of 1-s noting that where RF shock or burn might be possible, 6 or 30-min averaging times were no longer valid. In uncontrolled environments, the standard did not spell out an averaging time for induced or contact currents. None-the-less, the concern that even very momentary discharges can result in tissue damage seems to have influenced the very short averaging times specified for measurement of contact currents.

IEEE changed the averaging time for induced body currents in its 1999 standard [8] from the previous 1-s to 6 min for both controlled and uncontrolled environments. The same 100 and 45 mA limits for uncontrolled and controlled environments were retained, however applying these limits only over the 0.1 to 100 MHz frequency range in contrast with the ICNIRP contact current guidelines [5].

Significant changes to the IEEE contact current MPEs appeared in the 2005 IEEE standard [9]. Contact currents were now specified for two different conditions of contact, that of grasping contact (assumed to represent a contact area of 15 cm^2^) and that of touching contact (assumed to represent a contact area of 1 cm^2^). Despite an apparent disparity with the previously published standard, the averaging times for both induced and contact currents were set at 6 or 30 min, depending on whether the environment was controlled or uncontrolled (an apparent inconsistency in the text of the 2005 IEEE standard also stated that the averaging time was 6 min regardless of the environment). In 2010, the IEEE published an Amendment that specified ceiling limits for induced and contact currents but still contained the apparent typographical inconsistency [10]. More stringent values were placed on the MPEs for contact currents in the 2005 standard. Over the frequency range of 0.1–110 MHz (now increased from the previous upper frequency of 100 MHz), limits were specified as in Table 1.

**Table 1:** IEEE 2005 [9] contact current MPEs for the lower tier limits (action levels) and the upper tier limits (persons in controlled environments) over the frequency range of 0.1–110 MHz

Contact condition	Action level^a^ (mA)	Persons in controlled environments (mA)
Contact, grasp	–	100
Contact, touch	16.7	50

^a^The action level is the magnitude of contact current above which actions should be taken, such as instituting an RF safety program, to avoid exceeding the contact current limit

The standard emphasizes that “the grasping contact limit pertains to controlled environments where personnel are trained to make grasping contact and to avoid touch contacts with conductive objects that present the possibility of painful contact.” This language presumably refers to the issue of arcing that can occur with sufficiently high RF voltages. The common practice of “slapping” the contact can substantially reduce the burn hazard caused by potential arcing to the hand. It is noted that an action level for contact current relevant to a grasping contact is not specified since the presumption is that members of the general public cannot be adequately informed on how to effect a proper grasping contact to avoid excessive tissue heating.

Although the IEEE 2005 standard has not been revised since its publication (a revision of the standard is currently taking place), a more recent revision developed for the North Atlantic Treaty Organization (NATO) was produced in 2014 [11]. In this NATO version of the IEEE standard, limits for contact currents were made more complex in that they are were frequency dependent over the range of 0.1–110 MHz. Also, the averaging times of 6 or 30 min are used for induced currents but 1 s is used for the so-called touch condition for contact current. Table 2 shows the contact current limits from the 2014 publication (see note 3 for averaging times).

**Table 2:** IEEE 2014 [11] contact current MPEs for the lower tier (Zone 0, unrestricted environments where members of the general public may be present) and the upper tiers (Zone 1, restricted environments wherein persons are subject to an RF safety program and Zone 2, restricted experts only (REO) environment wherein persons are subject to an RF safety program and are deemed to be highly qualified for working in the vicinity of specific high intensity RF environments)

	Zone 0 (unrestricted environments)			Zone 1 (restricted environments)			Zone 2 (REO)
Frequency (MHz)	0.1–3	3–30	30–110	0.1–3	3–30	30–110	0.1–110
Contact, grasp	–	–	–	100	100 (f/3)^0.3^	200	250
Contact, touch	16.7	16.7 (f/3)^0.3^	33.4	50	50 (f/3)^0.3^	100	–

Tabulated value are rms values; *f* frequency in MHz

Limits apply to current flowing between the body and a grounded object that may be contacted by the person

The averaging time for determination of compliance is 6 min (Zone 1 and Zone 2) and 30 min (Zone 0) for induced currents, 1 s for touch current (Zone 0 and Zone 1), and 6 min for grasp contact current

Calculated values for personnel in Zone 0 and Zone 1 are capped at the 30 MHz values since there is insufficient data to extrapolate above 30 MHz

Light “brush” contact may result in arcs and shock and burn even at 50 mA and should be avoided, especially with long objects such as cranes or cables

For definition of each of the zones, see 3.1 in the IEEE standard

Restricted expert only access Zone 2 may be established only when mission essential and only when all personnel who are allowed access are expert on the particular system and informed that fingertip touch contact is to be avoided. Grasp is the appropriate method of contact

The ceiling values (temporal peak values as measured with accepted instruments) for induced current are 220 mA for Zone 0 (for a maximum duration of 75.3 s) and 500 mA for Zone 1 and Zone 2 (for a maximum exposure duration of 14.4 s)

In the NATO version of the IEEE standard, the terminology of “unrestricted” environments refers to environments where all persons are allowed access and where no individual will be exposed above the Zone 0 exposure reference level (ERL) and “restricted” environments that are accessible only to personnel who are aware of the potential for adverse health effects and methods to control their exposure from exceeding Zone 1 ERLs of the standard, respectively.

The standard specifies that the grasping contact limit pertains to restricted environments where personnel are trained to make rapid grasping contact and to avoid touch contacts with conductive objects that present the possibility of painful contact.

In regard to the issue of arcing, [11] continues the recommendation provided in the IEEE 2005 standard [9] that an open circuit RF voltage of 140 V is protective against arcing conditions for general public and personnel permitted in restricted environments.

In summary, the contact current limits reviewed here are generally based on current magnitudes that will limit specific absorption rates (SARs) in regions of small conductive cross section (such as the ankle and wrist where the highest local current density would exist) to less than the basic SAR restriction of the standard (basic restriction or BR). This has been examined in the context of RF hot spots, small regions of locally high strength electric fields, where the contact current magnitude required to achieve local SARs of 8 and 20 W/kg in the wrist [12] has been determined. The values 8 W/kg (averaged over 1 g of tissue) or 20 W/kg (averaged over 10 g of tissue in the extremities) correspond to the local BRs which were included in, at the time, the anticipated ANSI/IEEE standard for controlled environments [1]. In those analyses, a given current induced to flow within the body was used in conjunction with the conductive cross section of the wrist, some 11.1 cm^2^ [13] and tissue resistivity to assess current density and corresponding SAR. However, the matter of just how the current entered the body via contact was not explored and it is here that the more significant question of local heating at the surface of the skin lies. The contact current limits have not necessarily been based on the surface heating effect at the point of contact.

## Some past experimental work

The work of two different research groups was used in development the 2014 IEEE NATO standard [11] to revise the existing contact current limits. Rogers [14] first published data on individual perception and pain thresholds for contact currents in the high frequency (HF) band (3–30 MHz). The work was driven by concerns that personnel could experience high contact currents onboard navy ships in the vicinity of HF transmitting antennas. Subsequently, [13] measured body impedance and threshold contact currents that would result in perception and pain associated with localized surface heating. In examining these data for insights on the impact of RF contact currents, it is important to note differences in how the investigators approached the issue of making contact with an RF energized electrode.

In [14] individuals were tested by placing the back of the forefinger (moistened with saline solution) to an 18 mm diameter cylindrical rod electrode and adjusting the measured contact current to reach a level at which the subject perceived slight warming at the point of contact and when the temperature was such as to elicit withdrawal from the electrode (pain). The back of the finger was selected because it was found to be more sensitive than the front. Rogers [14] also made limited measurements with the front of the finger as opposed to the back of the finger and with a full grasping contact of the electrode. Results for 50 persons were reported suggesting that the approximate “average hazard threshold” current was 200 mA for the frequency band 2–20 MHz. Perception and let-go (pain sufficient to cause withdrawal from the electrode) thresholds were found to be about twice as great for front of the finger as for back of the finger contact. Rogers [14] reported that by firmly grasping the electrode, more than 500 mA could be tolerated for short periods without discomfort. It is significant to note that [14] reported that the finger was moistened with saline solution “to minimize variations in contact resistance.”

Other researchers [13] performed studies similar to that of [14] using a 15 mm diameter cylindrical rod electrode for testing of grasping current thresholds and square copper plate electrodes of either 144 or 25 mm^2^ area for contact with the back and front of the index finger. In all cases, for grasping or touch contacts, the skin was moistened with 0.9% saline solution to ensure a good electrical contact between the skin and the electrode. The data from [13], however, only included measurements up to 3 MHz, making it difficult to relate to most of their data. Nonetheless, [13] reported an average perception threshold touch contact current of about 40 mA from 100 kHz to 3 MHz. Further, their data indicated that the touch pain threshold current was only slightly greater than the perception threshold value, pain being reported after 10–20 s after sensation of perception of heating. It is not clear as to what accounts for the difference in reported perception and pain threshold contact currents between the two researchers. Obviously, additional data are needed to resolve some of the issues. But, notably, both investigations employed the use of saline solutions to stabilize the contact resistance between the electrode being contacted and the skin (i.e., thereby creating an artificial but relatively uniform contact resistance). The importance of this will be discussed later. A potentially useful insight accredited to [15] is the so-called spreading resistance of the tissue immediately beneath the electrode which was reported to be proportional to 1/√A where A is the electrode area in contact with the skin.

In summary, local application of RF currents can result in localized heating of the skin and, potentially, underlying tissues. Work to investigate threshold values of contact current that can be perceived by individuals as thermal warmth or pain have been determined under conditions of optimum electrical contact with the skin. As will be seen, such exposure conditions will result in the lowest tissue temperature increase for a given contact current or, conversely, the highest current thresholds for perception of heating.

## Technical considerations

Recently, a series of limited measurements of skin surface heating associated with RF contact currents has revealed factors relevant to better understanding the challenges of setting contact current limits [16]. The measurements consisted of applying contact currents of 100 mA through a round 1 cm^2^ disk electrode to skin and a synthetic tissue material at nine frequencies throughout the HF band. Using a thermographic camera, surface heating was observed from which high resolution (20 FPS) temperature/time data were obtained.

Several factors observed and described in [16] bear directly on the experimental basis for establishing contact current limits.

### Edge effects

The well known ‘edge effect’ of electrodes applied to the body surface can result in RF current density being concentrated at the periphery of a current carrying electrode [17, 18]. The practical impact of this phenomenon has been examined in detail in relation to the practice of electro-surgery where excessive heating associated with alternate site (current return electrode) RF burns is not uncommon [3]. A possibility that edge effects might occur in studies of electrode heating of the skin has been discussed in [19]. We argue that the electrode edge effect could be a factor in measurements of thresholds for thermal perception and pain. In [16] the presence of the edge effect was seen to be influenced by electrode contact resistance; with a highly conductive electrode–skin interface, the edge effect was more evident. When a significant portion of the contact current flows through a relatively smaller area than the total electrode surface, more intense heating of tissue will occur, thereby leading to a difference in human thermal perception thresholds if the edge effect is not present. An additional observation in [16] was that very small regions (order of 1–2 mm diameter) of higher conductivity in the skin can apparently result in micro-hot spots with extremely high local current densities and associated very intense localized heating.

### Contact resistance effects

Perhaps the most important factor related to heating in [16] was simply the resistance of the electrical contact between the electrode delivering the current and the skin. At 1.9 MHz, a contact current of nominally 100 mA was found to raise skin temperature to 45 °C in 18.8 s when the electrode was in contact with dry skin. When the electrode was coated with an electrically conductive gel, the same current took 131 s before the skin reached 45 °C. This observation shows that the contact resistance between the electrode and the skin surface plays a crucial role in skin heating for a given contact current. This is significant with respect to setting contact current limits in that the extent of a thermal hazard appears to be strongly dependent on the contact conditions at the time of exposure. It is noted that the two primary data sources used for development of the present contact current limits in the IEEE standard both ensured that contact resistance was minimized through the use of saline solutions to wet the skin surface prior to applying RF currents. In [16] it was also shown that the real part of the contact impedance between the disk electrode and skin was greatest at the lowest frequency, decreasing rapidly with increasing frequency. It was noted in [16] that a known and fixed force between the contact electrode and the skin was used to minimize variations in contact resistance. The nominal difference in contact resistance between the dry electrode and the gel coated (wet) electrode was measured by driving the electrode with an RF signal at 1.9 MHz and measuring the RF voltage across the electrode in conjunction with the current flowing through the electrode. Based on the relation R = V/I, the dry contact resistance was estimated at approximately 492 Ω while the wet contact resistance was approximately 146 Ω. This is a difference of 3.4 times and would be expected to be reflective of the higher resistance contact heating at a substantially greater rate. This higher heating rate is a function of the outermost layer of the epidermis for which the conductivity is least [20] but the conductivity at the surface is directly related to moisture content [21]. Skin dielectric properties and the significance of skin moisture content as it relates to bioimpedance has been studied by [22]. Clearly, for a given contact current, greater skin heating will be caused by higher contact resistance.

### Frequency effects

**Fig. 1:**
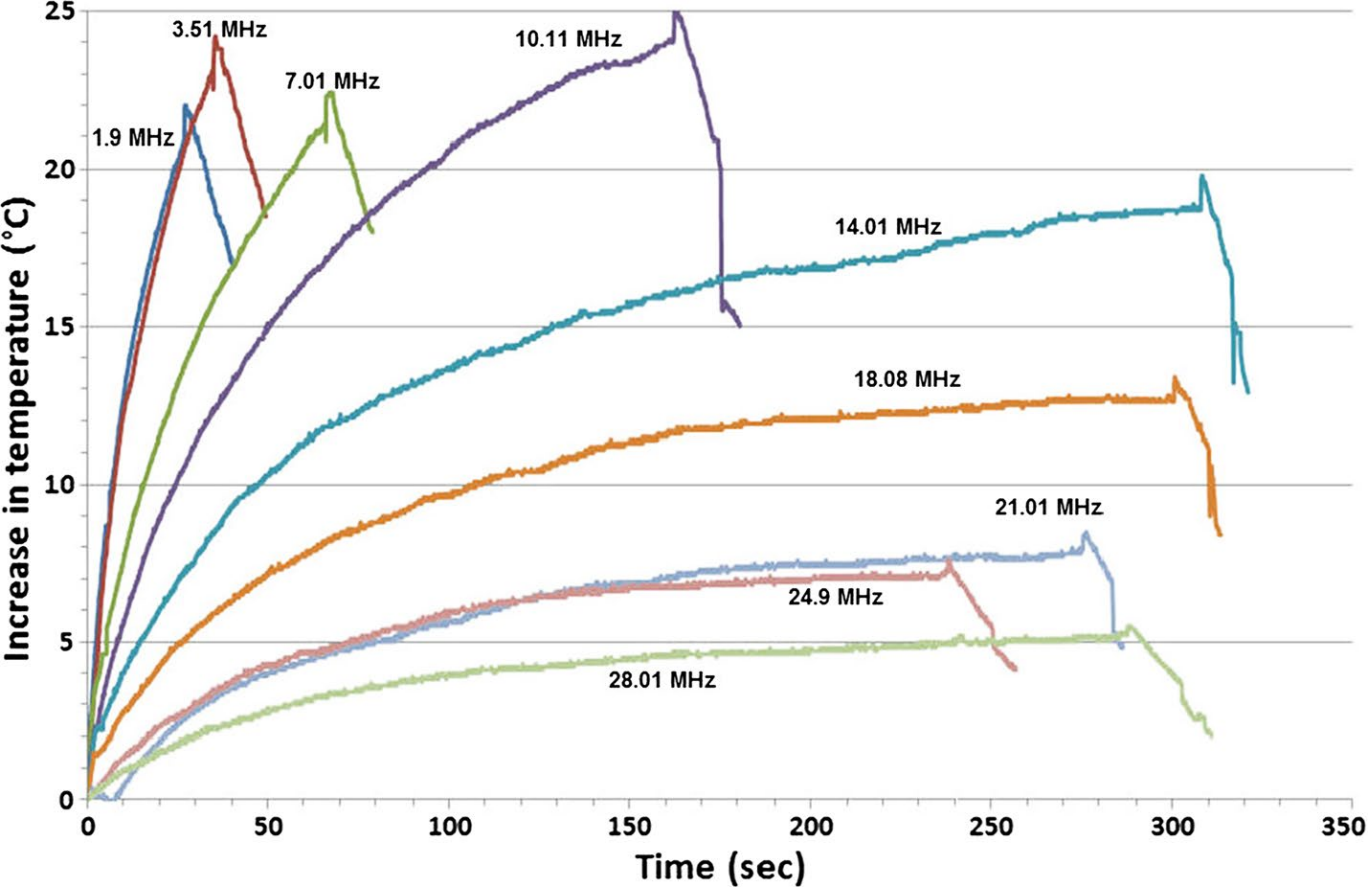
Maximum temperature on surface of synthetic tissue sample for 100 mA contact current at nine different HF band frequencies as a function of contact current flow duration with a dry contact electrode. The above curves were developed by application of RF current via a 1 cm^2^ round disk electrode placed on a sample of synthetic tissue and measuring maximum surface temperature with a thermographic camera at the rate of 20 samples/s. Contact current was terminated just prior to the peak value of temperature shown for each curve (Reproduced with permission from [16])

The threshold current for sensation of heating was observed in [14] to increase with frequency in the HF band. A similar trend was found in [16] when contact current was applied to a synthetic tissue over the HF band (1.90, 3.51, 7.01, 10.11, 14.01, 18.08, 21.01, 24.90 and 28.01 MHz) with a dry contact electrode. Figure 1 reproduced from [16] illustrates a decrease in heating rate at higher frequencies for the same applied contact current. Maximum temperatures varied based on the duration of current flow and the rate at which the synthetic tissue increased in temperature followed a pattern based on frequency. This pattern is potentially reflective of a change in the impedance of the contact with skin as well as the electrical characteristics of the synthetic tissue. The RF power deposited at the electrode is proportional to I^2^R where R is the resistance at the interface of the electrode with the skin and I is the contact current. These data were consistent with the casual observation with skin heating of the arm that a given contact current at higher frequencies was not as effective in heating the skin [16].

## Discussion

Regardless of whether RF current heating is of human skin in vivo or some alternative surrogate, the underlying influence of contact resistance must be recognized in relation to real-world contact current hazards. Assessing the suitability of a surrogate material for characterizing heating effects of RF contact currents will require further work. Regardless of the substitute for human skin, however, the matter of contact resistance of the application electrode must be appreciated in regard to local heating effects.

Some previous work used the perception of warming, not pain, as an endpoint [13] for quantifying response to contact current. However, safety standards are usually predicated on protecting against actual hazards and as such, pain is probably the best surrogate for indication of a potentially hazardous exposure. Contact current limits have been established in both the IEEE standard [9] and ICNIRP guidelines [5] to provide protection against known adverse health effects in the form of shock and RF burn hazards. IEEE states that for frequencies in the range of 3 kHz–5 MHz, contact current limits are designed to minimize aversive or painful electrostimulation and to protect against adverse heating. Hence, for the HF band, the presumed endpoint indicative of an adverse biological effect is tissue temperature that results in pain.

The literature provides a range of temperatures that reportedly result in the sensation of pain but, generally, a skin temperature of approximately 45 ± 1 °C seems to be taken as the nominal threshold for pain in many subjects [23]. However, the pain threshold varies among individuals and has been reported to vary with gender [24]. Heat pain thresholds have been found to be as low as 42 °C in the chest [25]. In another study, [26] reported that individuals characterized as “evening types” were significantly more sensitive to thermal pain than “morning types”. He reported a 2.9 °C higher threshold of skin thermal pain for morning types at the wrist (50.1 vs. 47.2 °C). Just what exact location within tissue is relevant to the sensation of pain has been examined [27] where nociceptors (the thermal sensors in the skin) were presumed to be located at a depth of some 50 μm; the outer most layer of the epidermis does not contain any thermal sensors, they lie just beneath that layer. This fact has been used by NASA in defining touch temperature limits where they state that such limits should be based on epidermal/dermal interface temperature at pain onset [28]. They argue that previous standards have incorrectly defined allowable object touch-temperature as the skin surface temperature limit which is overly conservative.

An issue that has not been studied in any depth relative to setting acceptable contact current limits is the matter of the so-called edge effect. From studies making use of electro-surgical equipment, the edge effect has been well known for many years [3, 4]. In [17], the issue of excessive heating, to the point of burning, was analyzed theoretically for a round electrode. They found that the enhanced peripheral current density in tissue at the edge of the electrode would lead to a substantially greater heating effect than directly beneath the electrode. In fact, in a separate paper [29] it was estimated that the local current density at the edge of the electrode is determined by about half of the total electrode current being collected by the outer 15% of the contact area. This implies that the peripheral current density could be as much as three times the current density under the electrode. This can result in as much as nine times the actual tissue heating based on the average rate under the electrode as a whole.

The edge effect was also studied by [18], finding very similar results. They found in their theoretical analyses that the local current density at the edge of the electrode exhibited a non-uniformity coefficient (ratio of maximum to minimum current density) of approximately 3.0 for the circular electrode and about 4.6 for a square electrode of the same area from corner to corner (with the corners exhibiting the greatest current density). Such results suggest that the edge effect could conceptually result in different thresholds for perception and pain from electrode heating across different researchers due to the differential heating effect at the edge of the electrode. Hence, caution should be used in interpreting perception and pain threshold data for use in setting RF contact current limits as the thresholds determined may exhibit a considerable variability depending on the exact technique used to apply the contact current. We would note that in the two principal research reports used as a basis for the IEEE NATO standard [11], contact current thresholds were assessed by placing the finger against the side of a round rod electrode as opposed to using a specific flat electrode having a known exact area.

In most research on RF burns, such as that associated with electrosurgical techniques, effort is made to minimize the resistive component of impedance between the application electrode and the skin (e.g. application of saline solution or conductive gel). The highly conductive contact between the skin and the electrode tends to minimize the thermal effect of RF currents when compared to dry, high impedance contacts. Highly resistive contacts can occur during work by individuals experiencing accidental contact with RF energized sources. Hence, while useful in gaining insight to the degree of heating damage that RF contact currents may represent, such data will not necessarily represent typical responses of individuals to highly localized skin heating. In [30] skin burns from electrosurgical currents were examined using porcine skin on intact pigs as a surrogate for human skin. They formulated a so-called “relative energy density factor” which was the product of the square of local current density (J) in amperes per square centimeter and time (t) in seconds and created categories of low, medium and high degrees of thermal skin damage based on visual examination of the burn sites. They reported that in the low range of damage, maximum skin temperatures were in the range of 38–47 °C with either no damage or a mild second-degree burn lying just beyond the electrode contact zone. The medium range of energy density factors were associated with maximum skin temperatures in the range of 49–55 °C with more substantial levels of thermal damage. The highest energy density factors corresponded to skin temperatures of 55–81 °C with the most severe burn effects. They noted that with skin temperatures less than 45 °C, no significant skin damage was produced corresponding to an energy density factor of 0.75 J^2^t. With a contact of area of 1 cm^2^, as used in [16], and an applied contact current of 100 mA, an exposure time of some 75 s would result in an energy density factor of 0.75 J^2^t. It is important to note that the energy density factors reported in [30] are based on visual examination for actual tissue damage ranging from erythema, through second-degree (reddened from cutaneous congestion and hemorrhage, adherent scabs of coagulum) to third-degree burns (yellow to brown, firm, dry). Further, the results reported by [30] where associated with good conductive contact between the electrode and the skin. Such categorization relates to tissue damage as opposed to a measure of perception or pain response and this difference could imply substantial differences in what an acceptable (safe?) contact current might be. For example, if protection against actual tissue damage is the appropriate criterion for a standard, then skin pain may be deemed as a conservative precursor to such damage. If pain itself is deemed the end point to avoid, then lower values of contact current will be appropriate.

Lastly, greater contact resistance is clearly related to lower thresholds for heat related adverse effects from RF contact currents. Interestingly, we would emphasize that an inverse relationship holds when determining thresholds of adverse reactions to low frequency currents that result in shock reactions. In the former, high contact resistance results in greater heating and a more conservative, lower current threshold for pain. In the case of shock thresholds, however, low contact resistance results in a more conservative, i.e., lower current threshold. We believe these observations suggest that the setting of contact current limits is more complicated than might appear. For instance, a fixed value of contact current can result in a wide range of biological response, depending on the conditions relevant at the moment of contact.

For contact currents associated with small area contact, localized heat development within the skin is crucially dependent on the effective resistance at the point of contact as well as exposure duration. Good electrical contact between the skin and an energized electrode results in substantially less heating while a high resistance contact, commonly associated with dry skin, results in increased heating, both with the same contact current. Further, for a given contact current, lesser heating occurs as frequency is increased within the HF band. For a given current, the temperature at the point of contact can asymptotically reach a plateau value where heat generation becomes balanced with heat dissipation such that a steady state temperature is achieved regardless of exposure duration. The influence of frequency on heating is most evident at the lower end of the HF band where the contact impedance exhibits a maximum value. Importantly, reports from [13] and [14] on contact currents made use of saline solution applied to the finger in their test subjects which implies that the contact current threshold values obtained were likely higher than what would have been observed under more realistic conditions in which dry contact could have happened. Overall, available data suggest that the challenge of setting technically based limits on RF contact currents is complicated by the relatively wide range in surface heating that can occur due to contact resistance and frequency.

Deciding on the basis for setting contact current limits is fundamental; is simply the perception of thermal warmth, possible pain associated with localized skin heating or actual tissue damage to form the basis for setting contact current limits? This question must be answered so that tissue heating data as reported by different researchers can be properly judged to establish adequate limits. Of special relevance to determining safe current levels, virtually all existing data on effects of contact currents have been acquired under optimal electrical contact conditions. Clearly, the setting of contact current limits based on an assumption of ideal electrical contact with RF energized objects will result in greater values of current being presumably acceptable. However, such an approach may not adequately protect against the underlying basis for limits for incidental contact with energized sources by all individuals under all possible conditions of contact. The wide range of contact impedance and contact duration during realistic exposure conditions leads to an inherent challenge. That challenge is rationalizing expected skin temperature with RF contact currents appropriate to both occupational exposures and exposures of the general public. Finally, while training of personnel on how to work in environments where excessive contact currents may be present is obviously appropriate, we would encourage careful consideration when developing guidance for avoiding RF contact current hazards based on the use of behavioral awareness such as methods for effecting contact with RF energized objects.

## Conclusions

Present limits on RF contact currents are based on the magnitude of the current with varying averaging times for these currents. In the 2005 version of the IEEE standard [9], averaging times were set at 6 min for the square of the contact current. A “touch” contact provision was not included prior to the 2005 IEEE standard. In the 2014 NATO version of the IEEE standard [11], touch contact current limits vary in three ranges of frequency between 3 and 110 MHz and are averaged over any 1 s. The extremely short duration averaging time for RF contact currents would appear to negate any kind of thermally related effect for the touch limits. In fact, the NATO touch limits are likely unrealistically conservative based on observations about heating from HF band contact currents appropriate to the touch condition.

Protection against thermal hazards associated with RF contact currents is likely most accurately related to controlling tissue temperatures. Temperature measurements in the workplace are, however, generally not practical. We recommend that future efforts to develop limits on contact currents include the following considerations:

1. The fundamental basis for the limits, perception of heating, thermal pain or skin damage from an RF burn; sensation of pain from high local temperatures would be consistent with the approach used by IEEE [9] in setting limits for contact currents at low frequencies wherein pain associated with neurostimulatory effects dominate.
2. Recognition of the practical conditions of real world exposures for contact currents; determining how contact is typically accomplished by a worker with RF energized equipment/objects and assessing the range of electrode–skin contact resistance that can be found in the workplace will identify relevant electrode contact areas and contact resistances for studying thresholds of reaction to contact currents.
3. Consistency of physical contact current application across multiple studies; threshold current data will be maximally uniform across different researchers if the same size and shape of electrode as well as contact force and contact impedance are used.
4. Frequency dependency of threshold data; when evaluating contact current thresholds from different researchers, common frequencies must be used to minimize uncertainties in reported results.

Finally, it is important to note that all contact currents, including those associated with touching, grasping or brushing contacts, will be subject to the same factors identified here.
